# The nociception level index (NOL) response to intubation and incision in patients undergoing video-assisted thoracoscopic surgery (VATS) with and without thoracic epidural analgesia. A pilot study.

**DOI:** 10.12688/f1000research.15279.1

**Published:** 2018-06-22

**Authors:** Laurent Bollag, Srdjan Jelacic, Carlos Delgado Upegui, Cynthia Wu, Philippe Richebe

**Affiliations:** 1 Department of Anesthesiology & Pain Medicine, University of Washington, Seattle, Seattle, Washington, 98195, USA; 2 Department of Anesthesiology & Pain Medicine, University of Montreal, Montreal, Quebec City, H1T 2M4, Canada

**Keywords:** intraoperative nociception monitoring, epidural analgesia, multiparametric nociception monitoring, lung surgery, regional anesthesia, general anesthesia

## Abstract

**Background: **The PMD100™ (Medasense Biometrics Ltd., Ramat Yishai, Israel) is a novel non-invasive nociception monitor that integrates physiological parameters to compute a real-time nociception level index (NOL) in the anesthetized patients. Thoracic epidural analgesia provides effective analgesia and improves surgical outcomes. Side effects include sympathectomy, hypotension, changes in skin temperature and a decreased cardiac accelerator fiber tone. The purpose of this pilot study was to evaluate changes in NOL values after incision in patients with and without epidural analgesia.

**Methods: **Half of the patients scheduled for Video-Assisted Thoracoscopic Surgery (VATS) received a thoracic epidural catheter, placed and tested 2h before surgery and activated prior to incision. The other half of the patients received i.v. fentanyl (1 mcg/kg) five minutes before incision. Anesthesia and analgesia were maintained in a standardized manner. NOL and heart rate (HR) were compared before and after the nociceptive stimuli intubation and skin incision.

**Results:** NOL significantly increased in all patients after intubation by 10.2 points (CI: 4.5-16.0; p=0.002) as well as HR by 9 beats per minute after intubation in all patients (CI: 3.3-15.6; p=0.01). After incision, in patients without epidural analgesia the NOL increased by 13.9 points (CI: 7.4-20.3; p=0.0001), compared to 5.4 points (CI: -6.3-17.1; p=0.29) in patients with epidural analgesia. HR did not significantly vary after incision in both groups. The area under the curve of delta NOL and delta HR variations after incision were significantly different (p<0.05) between groups and delta NOL variations were significantly different from baseline values but not the delta HR variations.

**Conclusions: **This pilot study suggests that the PMD100™ Monitor may be a useful tool to evaluate the efficacy of an intraoperative thoracic epidural analgesia.

**Clinical Trial Registry Number**: ClinicalTrials.gov record ID: NCT01978379 registered 10/25/2014.

## Introduction

To date, there are no standards to assess intra-operative nociception in a quantitative manner. The PMD100™ (Medasense Biometrics Ltd., Ramat Gan, Israel) is a non-invasive nociception monitor. The device integrates multiple physiological, parameters, including heart rate (HR), heart rate variability, photo-plethysmogram wave amplitude, skin conductance level, and number of skin conductance fluctuations, movements and their time derivatives to compute a real-time nociception index, called NOL. All data is measured by a single finger-mounted probe. A monitor displays the nociception level (NOL) expressed as an index. The data is computed every five seconds. The index ranges from 0–100: a NOL of 0 suggests a very low sympathetic activation equal to a nociception free state, while a NOL of 100 suggests high sympathetic activation, representing high nociception levels.

The exact details of the utilized technology can be found elsewhere ^1^.

Recent clinical trials reported that the NOL index might have a higher sensitivity and specificity than heart rate (HR) and mean blood pressure (MBP) to detect painful stimulations such as intubation, incision and tetanic stimulations in patients under general anesthesia ^2, 3^.

Thoracic epidural catheters and neuraxial administered local anesthetics provide excellent intra- and post-operative analgesia ^4^ and are frequently used in patients receiving thoracic surgery to improve post-operative pain and pulmonary recovery ^3^. However, epidural side effects include a dermatomal reduction of sympathetic tone and could therefore affect NOL measurements, which are based on autonomous nervous system variables.

The purpose of this pilot study was to evaluate changes in NOL values after incision for trocar insertion for video-assisted thoracoscopic surgery (VATS) in patients with and without epidural analgesia. This would suggest that the NOL index is a reliable parameter to assess the epidural analgesia in the anesthetized.

Secondary aims included changes in delta NOL index, in HR and delta HR values before and after two clinical stimuli, intubation and skin incision.

## Methods

### Subjects

After institutional review board approval (#44750-B) informed consent was obtained from 20 eligible women and men, 18 years of age or older and of American Society of Anesthesiology Physical Status I-III, scheduled for video-assisted thoracoscopic surgery (VATS) at the University of Washington Medical Center in Seattle, USA. The trial was registered with ClinicalTrials.gov on the 11/07/2013 record ID: NCT01978379, the study period was from January till December 2014.

Non-inclusion criteria included refusal, non-English speaking, chronic use of opioid analgesics, severe psychiatric disorder, history of previous thoracotomy, Body Mass Index (BMI)>40, and current beta-adrenergic blocking agent treatment. Inability to measure the NOL or HR led to exclusion from the data analysis.

### Anesthesia, monitoring & NOL Sampling

Patients in the *epidural group* (n = 10) received a mid-thoracic epidural catheter at either T7/8 or T6/7 dermatomal level, per surgical request. All other patients (n = 10) were considered as the *no epidural group*, again per surgical request. Randomization, due to surgical preference for epidural analgesia, was not possible.

After successful epidural placement, at least 2 hours before surgery, all catheters were tested with 3ml 1.5% lidocaine (45mg) with 15µg epinephrine added, to confirm epidural catheter tip location.

All patients received 1–2 mg intravenous (i.v.) midazolam for anxiolysis before being transferred to the operating room where standard anesthesia monitoring was applied, including a five-lead electrocardiography, non-invasive arterial blood pressure, continuous pulse oximetry, and a bispectral index monitor (BIS) (Medtronic, Mansfield, MA, USA. Philips Bispectral Index (BIS ^®^), BISx Power Link™, IntelliVue MP70, Philips, Netherland).

Additionally, the PMD100™ nociception monitor finger probe was connected to the middle finger on the blood pressure cuff free arm. NOL values were displayed following a 30 second calibration phase. The NOL data was recorded on a laptop using the Medasense biometric software. For each case, laptop times were adjusted to the time of the Anesthesia Information Management System (DocuSafe Version 7.2, Merge Healthcare, Chicago, IL, USA).

General anesthesia was standardized and induced with intravenous 1.5mg/kg lidocaine, 2 mcg/kg fentanyl, 1–2 mg/kg propofol, and 0.5mg/kg rocuronium. The time between i.v. administration of fentanyl and intubation was standardized for all patients and set at 5 minutes. Intubation was performed with a double lumen 37 or 39 Fr tube. Hypnotic depth was maintained with 1–1.5% end-expiratory sevoflurane concentration and was adapted to the age adjusted minimum alveolar concentration (MAC) at 1 to 1.2 to achieve target BIS values between 40–60. 100% inspired oxygen was used throughout the study period. Positive pressure ventilation mode was used in all patients. Hypotension, defined as a 20% decrease in systolic or diastolic blood pressure from the first measured values in the operating room or a mean arterial pressure below 65 mmHg, was treated with 100 µg phenylephrine bolus as needed. Then patients were repositioned laterally to facilitate the surgical approach to the lung cavity.

Ten minutes before skin-incision patients in the *epidural group* received an epidural bolus of 5ml 2% lidocaine (100mg), while patients in the *no epidural group* were administered an additional 1 mcg/kg fentanyl bolus, five minutes before incision. The study period ended five minutes after skin incision.

### Statistical analysis

NOL and HR were recorded every five seconds during the study period. Presented values span 90 seconds before and 190 seconds after intubation and incision, respectively.

**Figure 1.**
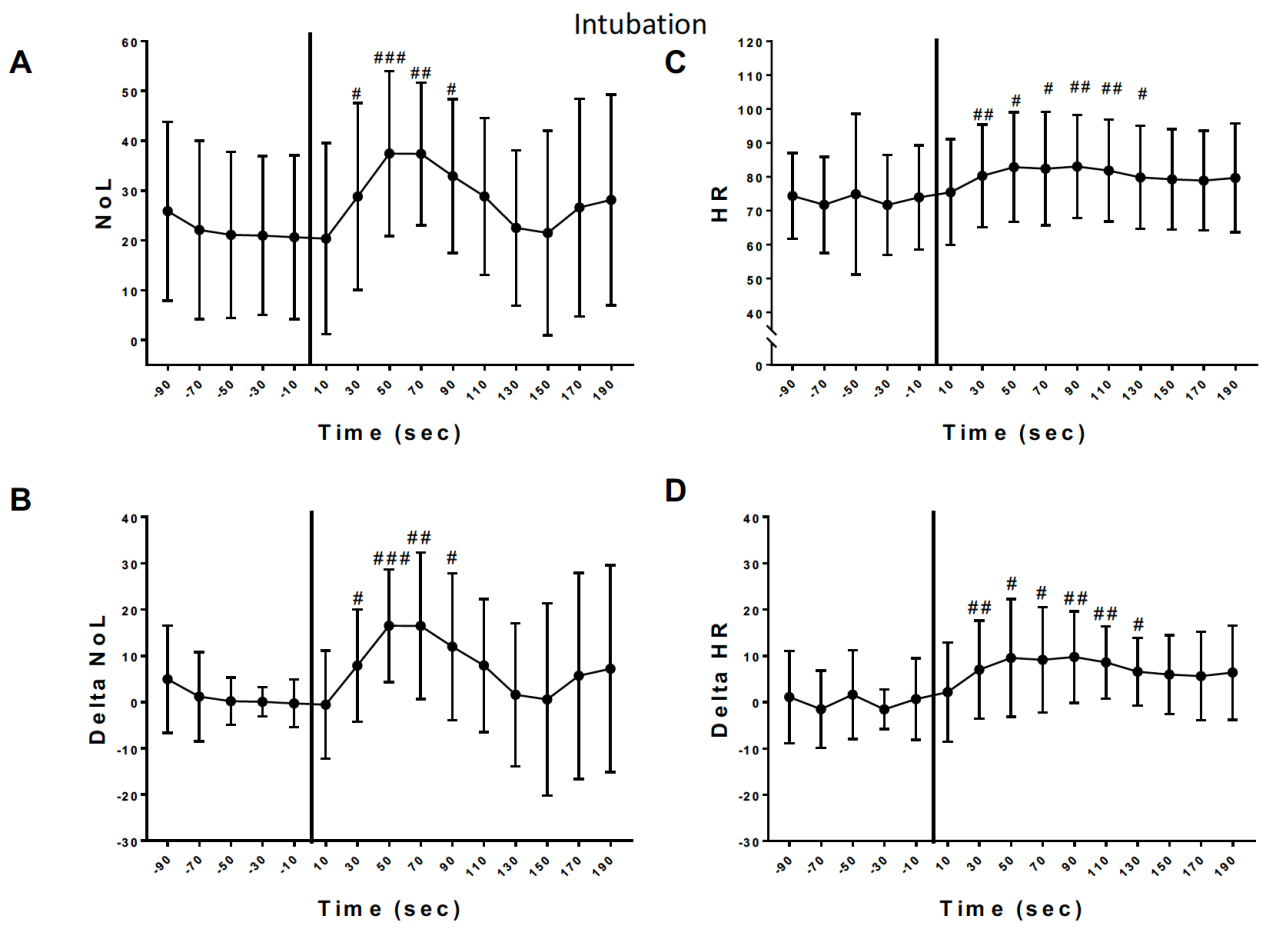
Variation of nociception level index (NOL) and heart rate (HR) after intubation. **A**) NOL variations after tracheal double-lumen intubation. There was no variation over time for the baseline NOL values prior to intubation (One Way ANOVA repeated measures, p=0.2312, F=1.523). There was a statistical significant variation in NOL absolute values after the tracheal intubation (One Way ANOVA repeated measures, p=0.0031, F=4,982). Dunnett’s multiple comparisons was used to compare each value to the **control** value at T minus 10sec. **B**) Delta NOL Baseline: There was no variation over time for the baseline delta NOL values prior to intubation (One Way ANOVA repeated measures: p=0.2312, F=1.523). There was a statistical significant variation in NOL absolute values after the tracheal intubation (One Way ANOVA repeated measures: p=0.0031, F=4.982). Dunnett’s multiple comparisons was used to compare each value to the **control** value at T minus 10sec. **C**) Heart Rate: Baseline without variation (One Way ANOVA repeated measures: p=0.5807, F=0.5187). After Intubation: significant variation in HR (One Way ANOVA repeated measures: p=0.0056, F=4,846). Dunnett’s multiple comparisons was used to compare each value to the **control** value at T minus 10sec. **D**) Delta Heart Rate: Baseline without variation (One Way ANOVA repeated measures: p=0.5807, F=0.5187). After Intubation: significant variation in delta HR (One Way ANOVA repeated measures: p=0.0056, F=4.846). Dunnett’s multiple comparisons was used to compare each value to the **control** value at T minus 10sec. For A, B, C and D significant differences with baseline values of each parameter are shown by: #: p<0.005, ##: p<0.001, ###: p<0.0001.

**Figure 2.**
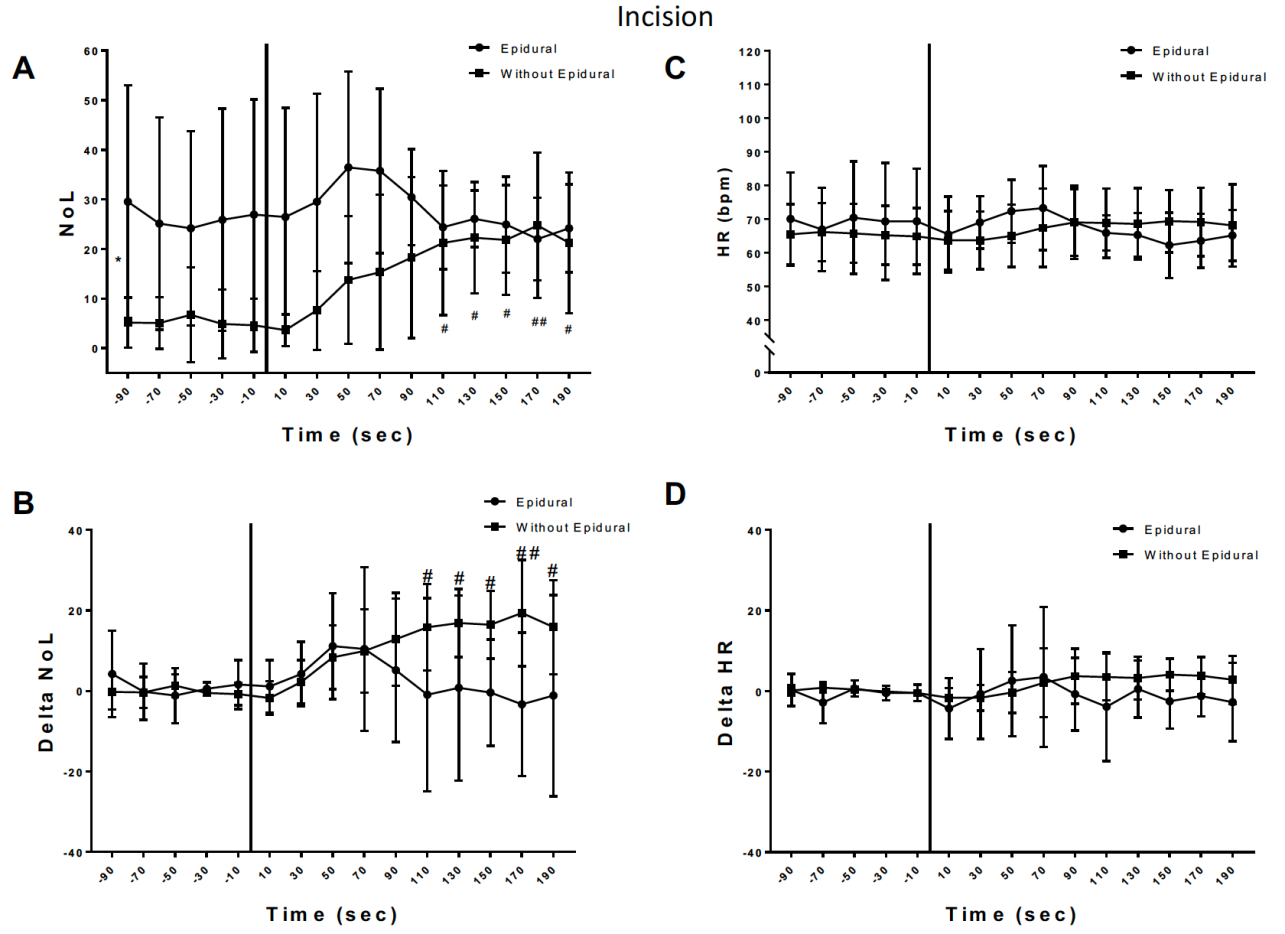
Variation of nociception level index (NOL) and heart rate (HR) after incision. **A**) NOL variations after incision. The baseline values (before incision) were analyzed using One Way ANOVA and showed no intra-group time effect (p=0.8148), meaning the NOL baseline values for each group were stable. Baseline NOL values were significantly different between groups before incision (p=0.0354, *). After incision: One Way ANOVA for repeated measures showed no significant difference in NOL values after incision in the *epidural* group. In the *no-epidural* group, one way Anova analysis and Dunnett’s multiple comparisons showed a significant increase of NOL values after incision (#, ##). Two Ways Anova (Time x Epidural) showed an Interaction (p=0.0015) and Bonferroni’s multiple comparisons showed a significant difference between *epidural* versus *no- epidural* at 10sec, 30sec and 50sec after incision (p<0.05). **B**) Delta NOL baseline values showed no difference between groups and were stable Two Ways Anova (Time x Epidural): Interaction p=0.1048, Epidural p=0.5891, Time p=0.4010). After incision, delta NOL increased significantly in the *no-epidural* group (Dunnett’s multiple comparison, p<0.05; #, ##) but not in the *epidural* group. Bonferroni’s multiple comparison between *epidural* versus *no-epidural* showed no statistical significant difference. **C** and **D**) HR or Delta HR baselines were stable prior to incision (One Way Anova, no time effect in each group) and not significantly different between groups. After incision, HR did not significantly change in both groups when compared to baseline values (One Way ANOVA, Dunnett’s multiple comparison for time, no time effect in each group). Bonferroni’s multiple comparison comparing epidural versus without epidural showed no significant difference between two group after incision for HR and delta HR values. For A, B, C and D significant differences with baseline values of each parameter are shown by: #: p<0.005, ##: p<0.001.

For *“Pre-stimulation” NOL and HR values*, the values at “-10 seconds” before an event were used, after an Anova one-way analysis showed that pre-stimulation NOL and HR values were stable and did not vary during the preceding 90 seconds period (see Figure 1 and Figure 2).

For “ *Post-Stimulation*” values NOL or HR were averaged every 20 seconds for 180 seconds after an event. In Figure 1 and Figure 2, the dots represent values averaged over 20-second periods. NOL and HR values are presented as means and standard deviations.

Pre and post-stimulation data was compared using Anova two-ways analysis and post-hoc analysis, Dunnet’s test was performed to evaluate the changes over time for each parameter, as well as the difference between groups ( Figure 2).

Delta NOL/HR signals were calculated by subtracting the baseline NOL and HR values from the signal. The baseline value was defined as the average NOL or HR value over the last 60 seconds before an event (-60 to 0 sec).

For delta NOL and delta HR, the area under the curve (AUC) was calculated in the time window from the stimulus, at 0 sec, to 180 seconds and compared with the Student t-Test.

A p-value of less than 0.05 was considered significant to reject the null hypothesis, which was that average NOL and HR values before and after the two events as well as their changes in patients with and without thoracic epidurals would not change.

For this pilot study, no power calculation was made to determine the number of subjects to be included. This number of subjects per group was arbitrarily set at 10.

The Statistical analysis was performed using IBM SPSS Statistics for Mac version 24.0 (IBM Corp., USA).

## Results

After consent was obtained, 20 subjects were included into this pilot study. Due to a technical fault with the study computer and broken cable, only the data of 16 patients could be analyzed; 8 with and 8 without epidural catheters. Demographic data are presented in Table 1.

**Table 1.** Demographic data.

	Epidural Group *(n=8)*	No Epidural Group *(n=8)*
**Age** (years)	54.6	51.4
**Height** (cm)	169.9	177.9
**Weight** (kg)	79.4	101.7
**BMI** (kg/m ^2^)	27.4	32.1
**ASA - mode**	3	3
**ASA - range**	1 – 3	1 – 4

BMI – Body mass index, ASA – American Society of Anesthesiologists physical status score.

During the study periods (see methods), no vasoactive drugs such as phenylephrine were administered. After intubation, the NOL and HR increased significantly in all patients by 11.3 points (CI: 2.7–19.9; p=0.013) and 9.4 bpm (CI: 3.3–15.6; p=0.0105), respectively in all patients compared to the baseline value (average of values over 60 seconds before event). NOL and delta NOL significantly increased after intubation for 90 seconds ( Figure 1 A, B). After intubation, NOL values increased 90% compared to baseline values. HR and delta HR also significantly increased after intubation for 130 seconds ( Figure 1 C, D). HR increased only by 12% after intubation when compared to baseline values.

After skin incision mean NOL values in the *no-epidural* group increased by 13.9 points (CI: 7.4–20.3; p=0.001) compared to 5.4 points (CI: -6.3–17.1; p=0.29) in the epidural group. The mean difference between *no-epidural* and *epidural* groups was 8.4 points (CI: -3.7–20.6; p=0.15). After the incision, NOL and delta NOL values significantly increased until 190 seconds in the *no-epidural* group, in the *epidural* group no significant change was observed ( Figure 2A, B).

The skin incision stimulus did not increase HR and delta HR significantly in both groups and the mean difference in HR increase between groups was only 0.8 (CI: -7.6–9.2; p=0.84) ( Figure 2C, D).

The areas under the curve, calculated for delta NOL and delta HR after the incision, showed a significant lower delta NOL AUC in the *epidural* group than the *no-epidural* group ( Table 2). AUC calculated for delta HR after incision did not show any significant difference between the groups.

**Table 2.** Mean area under the curve (AUC) for delta nociception level index (NOL) index and delta heart rate (HR) after incision stimulus.

AUC	Delta NOL	Delta HR
Epidural	3.22	-1.26
Without Epidural	11.12 ^*^	1.84

* represents p≤0.05, Student t-Test.

Nociception level index (NOL) Dataset Click here for additional data file. Copyright: © 2018 Bollag L et al. 2018 Data associated with the article are available under the terms of the Creative Commons Zero "No rights reserved" data waiver (CC0 1.0 Public domain dedication).

## Discussion

This is the first study assessing the feasibility of NOL to evaluate the quality of epidural analgesia during a nociceptive stimulation under general anesthesia.

In this pilot study, the level of nociception (NOL) assessed with the PMD100™ pain monitor as well as HR, significantly increased in all our patients after intubation ( Figure 1) as previously reported by other authors ^2, 3^. This significant increase in NOL, Delta NOL, HR and Delta HR lasted for 1.5 to 2 minutes after the stimulus, despite a moderate amount of i.v. fentanyl dose (1mcg/kg) was administered 5 minutes before intubation. This demonstrates that intubation remains a strong, detectable nociceptive stimulus under general anesthesia with appropriate fentanyl analgesia.

In our study, NOL index increased by 90% after intubation whereas HR increased by only 12% for the same stimulus. This finding aligns with previous reports that the NOL index might have a better sensitivity in detecting noxious stimulus such as an intubation ^2, 3^.

Skin incision followed by the first trocar insertion and endoscope placement for the VATS procedure caused a significant NOL index increase in patients without epidurals, despite the standardized fentanyl administration prior to incision. In patients with a prior to incision activated epidural catheter in place, NOL values did not significantly increase after incision ( Figure 2A, B), while the HR did not significantly vary in both groups after incision ( Figure 2C, D) emphasizing the NOL’s higher sensitivity and specificity to detect nociceptive stimuli ^2, 3^. The observed smaller variations of the NOL index or the delta NOL after incision in the epidural group is likely caused by effective epidural analgesia and successful attenuation of the nociceptive autonomous response caused by the skin incision ^5^, hence they might be a good quantitative parameter to assess the quality of analgesia provided by an epidural analgesia.

Mean NOL values before skin incision were different between groups. A previous study found the threshold for nociception to be around a NOL index of 12 while values around 20 were associated with mild pain. The authors suggested a NOL value of 16 to be the threshold for pain detection under general anesthesia ^2^. In our study, patients were subjected to many types of stimuli after the induction of general anesthesia: manual ventilation, intubation, lateral positioning which all together can induce a small amount of pain or discomfort and might explain why the basal threshold of NOL was higher (20.3 +/-18 in the epidural group) compared to values reported in previous studies ^2, 3^ when patients were left at rest under general anesthesia.

This is particularly true in the *epidural* group as they did not receive supplementary doses of fentanyl as opposed to the *no-epidural* group (see methods). Because the epidural catheter was placed 2 hours before surgery and only a small (test) dose was injected, no effect of the epidural could be expected before intubation.

We found no difference in NOL values between the *epidural* and *no-epidural* group before intubation ( Dataset 1 ^6^). The lower NOL values of the *no-epidural* group before skin incision are likely caused by an analgesic effect of the intravenous fentanyl bolus (1 mcg/kg), given 5 minutes prior, questioning the predictive value of the monitor. Fentanyl yields a rapid onset of systemic analgesia, whereas epidural analgesia is dermatomal, only.

Monitors of nociception assess single or multiple changes of the autonomous nervous system, including HR and its variability, skin vasomotor reflex and conductance, and photoplethysmogram ^7– 10^. Some literature suggests that multi parametric indices are more sensitive compared to single parameter devices to detect mild and moderate noxious stimulation ^1, 11, 12^. There is no literature to date evaluating the effect of regional anesthesia on the variation of indexes after painful stimulus offered by these monitors.

In an obstetric population, lumbar epidurals were found to increase HR variability due to an increase in parasympathetic activity after epidural analgesia ^13^. Another study found that a neuraxial blockade reduced low-frequency power and high-frequency power of HR variability, suggesting a total decrease in autonomic activity ^14^.

Epidural autonomous nervous system blockade effects include dermatomal sympathectomy, hypotension, changes in skin temperature regulation ^15, 16^, decrease of cardiac accelerator fibers tone, and a slight reduction in heart rate ^17, 18^. Theoretically, all these changes could affect nociception measurements, such as the NOL.

Mid-thoracic epidural analgesia (T5-T11) inhibits efferent sympathetic preganglionic outflow, causing vasodilatation of the highly compliant splanchnic bed in a dose dependent manner that leads to a decrease of systemic arterial pressure because of venous pooling of blood in this region ^13, 19^. Additionally, the relative hypovolemia, secondary to the epidural sympathectomy-mediated vasodilatation might cause a physiological tachycardic response, potentially increasing NOL values in the epidural group; we did not observe this in our study.

We recognize several limitations in our study: a double blind randomized study design might have been a better choice, but this would have added complexity. This pilot study aimed at whether epidural analgesia can be detected by NOL in patients under general anesthesia in order to design stronger studies. Further, all data were electronically recorded, hence NOL values could not be influenced by the research, anesthesia or surgical teams. We are presenting the results of small pilot and feasibility study, future larger studies are warranted to evaluate if the NOL is useful for titration of epidural local anesthetics during combined general-epidural anesthesia.

In summary, this is the first study looking at the feasibility of assessing the NOL index in patients under general anesthesia and thoracic epidural analgesia and its ability to assess intra-operative epidural analgesia.

## Ethical standards

All procedures performed in studies involving human participants were in accordance with the ethical standards of the institutional and/or national research committee and with the 1964 Helsinki declaration and its later amendments or comparable ethical standards. This study was approved by the local ethical committee on the 24 ^th^ of June 2014 (Human Subjects Division, University of Washington, Seattle, WA USA. Chairperson: Jane Hitti, MD. Approval # 44750)

## Data availability

The data referenced by this article are under copyright with the following copyright statement: Copyright: © 2018 Bollag L et al.

Data associated with the article are available under the terms of the Creative Commons Zero "No rights reserved" data waiver (CC0 1.0 Public domain dedication). 

Dataset 1: Nociception level index (NOL) Dataset 10.5256/f1000research.15279.d207164 ^6^
