# Supplementary material for: The nociception level index (NOL) response to intubation and incision in patients undergoing video-assisted thoracoscopic surgery (VATS) with and without thoracic epidural analgesia. A pilot study
Source: F1000Res. 2018 Jun 22;7:875. [Version 1] doi: 10.12688/f1000research.15279.1 (PMC6039929; doi:10.12688/f1000research.15279.1)
Supplement: Peer review history

# Peer review: Referee response for version 1

**DOI:** 10.5256/f1000research.16645.r35366

**Published:** 2018-07-09

**Authors:** Georges Daccache

**Affiliations:** 1 Department of Anesthesia and Intensive Care, University of Caen Normandy, Caen, France

This observational pilot study was designed to evaluate the feasibility of monitoring the autonomic nervous system response to intraoperative stress (intubation and skin incision), by a novel and unique multi-parameter device, the nociception level index (NOL) compared to the heart rate response (HR) under sevoflurane general anesthesia for VATS.

 For the skin incision, the authors separated the patients in 2 groups of 8 each: 100 mg lidocaine bolus through an epidural catheter versus 1 mcg/kg intravenous fentanyl. The authors showed that NOL variations were more sensitive than HR variations in detecting the skin incision stress.

 This pilot study confirmed the reliability of monitoring by the NOL the autonomic response after the two most common intraoperative stresses (intubation and skin incision).

 Despite several limitations that have been well-discussed by the authors (not randomized, high basal NOL values in the epidural group), these encouraging results have to be confirmed by future larger studies and compared to other available nociception monitoring devices (SPI, Analgesia Nociception Index) in various clinical settings.

 Future studies should also focus on the clinical impact of monitoring nociception by the NOL in terms of postoperative pain and analgesic consumption.

I have read this submission. I believe that I have an appropriate level of expertise to confirm that it is of an acceptable scientific standard.

# Peer review: Referee response for version 1

**DOI:** 10.5256/f1000research.16645.r35367

**Published:** 2018-07-09

**Authors:** Emmanuel Boselli

**Affiliations:** 1 University of Lyon, Lyon, France; 2 Groupement hospitalier Nord-Isère, Centre hospitalier Pierre Oudot, Bourgoin-Jallieu, France

The authors have performed a study aiming at assessing NOL response to intubation and incision in patients undergoing video-assisted thoracoscopic surgery with or without epidural analgesia. The study followed a quasiexperimental non-randomized design. In total, 20 patients were included, and 16 were analyzed (8 in each group). They observed that NOL, ∆NOL, HR and ∆HR increased significantly in all patients after intubation. After skin incision, NOL and ∆NOL increased significantly in the epidural group, whereas no difference was observed for HR and ∆HR. The AUCs for ∆NOL and ∆HR after stimulus during 180 s were calculated. A significant difference was observed for ∆NOL AUC in patients with or without epidural analgesia. This was not observed for ∆HR. The authors conclude that NOL may be useful to evaluate the efficacy of an intraoperative thoracic epidural analgesia.

 Although of interest, there are some concerns regarding this study.

**Major Concerns:**

1. The authors should emphasize the fact that this study was a non-randomized quasiexperimental study performed on a small amount of patients (8 per group). Although some variations were observed, the results should be considered with caution and confirmed by larger trials.
2. Could the authors explain why ANOVA for repeated measures (and corresponding post hoc tests) were not performed for all time by group interactions ? It is unclear to me why no intergroup analysis was assessed (epidural vs no epidural) during time for intubation.
3. The AUC is somewhat confusing and should be better described (and referenced). What is exactly the information provided by this parameter? Moreover, this study doesn't provide any threshold for NOL or ∆NOL variation to assess any outcome. Could the author better explain how these variations may be useful to modify anything in the anesthesia protocol?

**Minor Concerns:**

1. Microgram should be labeled µg , not mcg, in the entire manuscript. Please correct.
2. Seconds should be labeled s, not sec, in the entire manuscript and figures. Please correct.
3. Table 2: please provide estimates of dispersion for AUC (95% CI, SD,...) and present data with groups in columns and values (∆NOL and ∆HR in rows).

I have read this submission. I believe that I have an appropriate level of expertise to confirm that it is of an acceptable scientific standard, however I have significant reservations, as outlined above.

# Peer review: Referee response for version 1

**DOI:** 10.5256/f1000research.16645.r35368

**Published:** 2018-07-02

**Authors:** Jean Pierre Estebe

**Affiliations:** 1 Department of Anesthesiology, Intensive Care, and Pain Medicine, University of Rennes, Rennes, France

The authors provide pilot study which try to evaluate the interest to monitor the anesthesia and surgical stresses by the NOL index (multiple physiological parameters). It was a prospective observational study with patients scheduled for video-assisted thoracoscopic surgery with or without thoracic epidural analgesia (function of patient preference). The comparison was made between the NOL and the usual cardiovascular response (HR, and MBP). During the first step the authors compare the variations during and just after the tracheal intubation under regular anesthesia protocol (lidocaine, fentanyl, propofol then sevoflurane, and rocuronium) under BIS monitoring. During the second step; similar evaluation was performed during the surgical incision (epidural 5mL lidocaine 2% Vs. bolus 1mcg/mL fentanyl). The authors reported a more sensitive and reliable NOL variations than HR variations (with or without sympathetic protection; i.e. intubation Vs. thoracic surgical incision).  

 My major remark is just based on semantic reflection. Instead of a discussion of intra-operative nociception it could be better to discuss the cardiovascular response to the stress, or in a more general way to the sympathetic / para sympathetic balance assessment to the anesthetic stress (intubation) and surgical stress (skin incision in this study).

 Minor Points:

 The authors must clarify the procedure used to control the epidural catheter tip location (ephedrine is just for the accidental IV administration) and his true efficacy (postoperative pain evaluation; not only by the lack of supplementary intraoperative doses of fentanyl). Table 1 results must be expressed with variations (i.e. +/- SD). It is a pity that the postoperative pain evaluation was not reported, and correlate or not, with NOL variations (or with the AUC of NOL). 

 Despite several limitations in the study reported by the authors (not double-blind randomization); the concept proof of reliable monitoring by the NOL index is confirmed in this pilot study. Unfortunately, the authors did not try to correlate these NOL variations with the level of postoperative pain (or analgesic consumption).

I have read this submission. I believe that I have an appropriate level of expertise to confirm that it is of an acceptable scientific standard.

# Peer review: Referee response for version 1

**DOI:** 10.5256/f1000research.16645.r35364

**Published:** 2018-06-27

**Authors:** Sarah Saxena

**Affiliations:** 1 Department of Anesthesia, Free University of Brussels, Brussels, Belgium

**Summary:**

 This pilot study evaluated the efficacy of an intraoperative thoracic epidural analgesia by using a multi-parameter device, the nociception level index (NOL).

 The authors offer an interesting perspective on the use of a multi-parameter intra-operative nociception device in patients undergoing VATS.

**Comments:**

 Abstract:

 please mention in the conclusion that more studies are needed.

 Introduction:

 please mention that NOL is the only nociception monitor currently available combining several parameters (in contrast to for example ANI; SPI)

 Discussion:

 In the limitations, you state that randomization would have added complexity. Can you please explain this?

 Please mention in the conclusion that future large studies are warranted.

I have read this submission. I believe that I have an appropriate level of expertise to confirm that it is of an acceptable scientific standard.
